# ANK3 rs10994336 and ZNF804A rs7597593 polymorphisms: genetic interaction for emotional and behavioral symptoms of alcohol withdrawal syndrome

**DOI:** 10.1186/s12888-024-05787-z

**Published:** 2024-05-03

**Authors:** Guanghui Shen, Li Chen, Yanlong Liu, Qi Zhu, Yimin Kang, Xinguang Luo, Fan Wang, Wei Wang

**Affiliations:** 1 Key Laboratory of Psychoneuroendocrinology, Wenzhou Seventh People’s Hospital, Wenzhou, 325006 China; 2 School of Mental Health, Wenzhou Medical University, Wenzhou, 325035 China; 3 Psychosomatic Medicine Research Division, Inner Mongolia Medical University, Hohhot, China; 4 Department of Psychiatry, Yale University School of Medicine, New Haven, CT 06510 USA; 5 Beijing Hui-Long-Guan Hospital, Peking University, Beijing, China; 6 Zhejiang Provincial Clinical Research Center for Mental Disorders, The Affiliated Wenzhou Kangning Hospital, Wenzhou Medical University, Wenzhou, China

**Keywords:** Alcohol withdrawal syndrome, *ANK3*, *ZNF804A*, Gene-gene interaction

## Abstract

**Objective:**

Alcohol withdrawal syndrome (AWS) is a complex condition associated with alcohol use disorder (AUD), characterized by significant variations in symptom severity among patients. The psychological and emotional symptoms accompanying AWS significantly contribute to withdrawal distress and relapse risk. Despite the importance of neural adaptation processes in AWS, limited genetic investigations have been conducted. This study primarily focuses on exploring the single and interaction effects of single-nucleotide polymorphisms in the ANK3 and ZNF804A genes on anxiety and aggression severity manifested in AWS. By examining genetic associations with withdrawal-related psychopathology, we ultimately aim to advance understanding the genetic underpinnings that modulate AWS severity.

**Methods:**

The study involved 449 male patients diagnosed with alcohol use disorder. The Self-Rating Anxiety Scale (SAS) and Buss-Perry Aggression Questionnaire (BPAQ) were used to assess emotional and behavioral symptoms related to AWS. Genomic DNA was extracted from peripheral blood, and genotyping was performed using PCR.

**Results:**

Single-gene analysis revealed that naturally occurring allelic variants in *ANK3* rs10994336 (CC homozygous vs. T allele carriers) were associated with mood and behavioral symptoms related to AWS. Furthermore, the interaction between *ANK3* and *ZNF804A* was significantly associated with the severity of psychiatric symptoms related to AWS, as indicated by MANOVA. Two-way ANOVA further demonstrated a significant interaction effect between *ANK3* rs10994336 and *ZNF804A* rs7597593 on anxiety, physical aggression, verbal aggression, anger, and hostility. Hierarchical regression analyses confirmed these findings. Additionally, simple effects analysis and multiple comparisons revealed that carriers of the ANK3 rs10994336 T allele experienced more severe AWS, while the *ZNF804A* rs7597593 T allele appeared to provide protection against the risk associated with the *ANK3* rs10994336 mutation.

**Conclusion:**

This study highlights the gene-gene interaction between *ANK3* and *ZNF804A*, which plays a crucial role in modulating emotional and behavioral symptoms related to AWS. The *ANK3* rs10994336 T allele is identified as a risk allele, while the *ZNF804A* rs7597593 T allele offers protection against the risk associated with the *ANK3* rs10994336 mutation. These findings provide initial support for gene-gene interactions as an explanation for psychiatric risk, offering valuable insights into the pathophysiological mechanisms involved in AWS.

**Supplementary Information:**

The online version contains supplementary material available at 10.1186/s12888-024-05787-z.

## Introduction

Alcohol use disorder (AUD) is one of the most common mental disorders globally, with high prevalence rates [1, 2]. AUD is characterized by repeated episodes of withdrawal and relapse, leading to resumed drinking. Alcohol withdrawal syndrome (AWS) occurs when long-term ethanol consumption is stopped and is characterized by symptoms such as hyperactivity of the central nervous system and autonomic nervous system, including tremors, nausea, vomiting, irritability, and anxiety [3]. Mood disorders related to AWS significantly contribute to AUD relapse [4], highlighting the importance of treating AWS as part of relapse prevention and intervention strategies for AUD [5, 6]. The emotional and behavioral symptoms associated with AWS, such as pronounced anxiety and aggression, significantly contribute to the distress experienced during withdrawal [7]. These symptoms compound the physiological perturbations of withdrawal and adversely affect the psychological equilibrium of those attempting to cease alcohol use. During withdrawal, negative emotional and behavioral symptoms such as anxiety, anger, and aggressive behavior reinforced, leading to a resumption of excessive drinking as a way to alleviate these symptoms [7]. Existing literature on AWS suggests prevention and intervention measures based on previous behavioral and neurological research on AWS. However, there is considerable variability among patients with AWS, making it a complex condition that requires careful evaluation for effective treatment. Recent studies on AUD have explored the roles of different gene receptors in AUD treatment [8], and animal studies have shown the influence of genetics on the severity of alcohol withdrawal syndrome after alcohol abstinence [9]. Yet, the specific genes that influence the severity of alcohol withdrawal are largely unknown. Since the development of alcohol withdrawal syndrome after abstinence is likely due to alcohol-induced neuronal damage in the brain [10–12], genes associated with brain neurons, such as *ANK3* and *ZNF804A* [13, 14], may have unknown mechanisms of action in alcohol withdrawal syndrome. Therefore, this study selected the *ANK3* and *ZNF804A* genes as candidates to analyze their genetic effects on AWS, aiming to contribute to a better understanding of neuroadaptive processes and provide insights into preventing substance dependence relapse.

*ANK3* is a major neuron-enriched gene involved in anchoring voltage-gated sodium channels to nodes of Ranvier and maintaining connections between axonal membranes and myelin rings [15]. Its primary function in the brain is the formation and maintenance of neuronal axon initial segments (AIS) [16, 17]. Pathogenic variants in *ANK3* are associated with various neurological disorders, including bipolar disorder [18], schizophrenia [19], and autism spectrum disorder [20]. Recent imaging genetics studies have demonstrated that the *ANK3* rs10994336 risk variant is associated with reduced white matter integrity, impaired decision-making, and increased risk-taking, which is a trait associated with increased aggression [21]. Furthermore, functional genomics analysis has revealed an association between alcohol consumption and *ANK3* gene expression, with low ethanol concentrations leading to downregulation of *ANK3* expression [22]. Investigating the effect of *ANK3* genetic polymorphisms on the severity of AWS-related psychiatric symptoms would be interesting. However, the impact of a single genetic polymorphism on AWS-related mood disorders is limited. Therefore, we aim to identify another target gene for interaction analysis.

*ZNF804A* is the first gene to reach genome-wide significance for both schizophrenia and bipolar disorder [23], although the exact function of its protein products is currently unknown. Recent studies have shown that *ZNF804A* plays a crucial role in controlling synapse formation, neurite proliferation, neuronal migration, and central nervous system development [24, 25]. *ZNF804A* alleles are associated with changes in neural activity, synaptic connectivity in healthy subjects, and neuroanatomical alterations in white and gray matter in several brain subregions [26, 27]. Our previous research demonstrated that the *ZNF804A* rs1344706 allele is associated with impulsivity in AUD patients [28]. Another SNP, rs7597593, in *ZNF804A* has been reported to interact with antidepressants to improve depressive symptoms, indicating a moderation effect of this SNP in the pathophysiology of depression [29]. Moreover, *ZNF804A* genetic polymorphisms (rs1344706 and rs7597593) have been associated with impaired decision-making in heroin abusers [30]. Both *ANK3* and *ZNF804A* are candidate genes associated with schizophrenia and bipolar disorder based on genome-wide association studies [21]. Therefore, in the present study, we selected *ANK3* and *ZNF804A* as candidate genes to examine the influence of genetic variation on AWS-related mood and behavior.

Genetic variations in ANK3 have been implicated in the alteration of neuronal functions. In AWS, these variations may influence the neuroadaptive processes disrupted by alcohol withdrawal, potentially impacting symptom severity and recovery. Similarly, Variants in ZNF804A are associated with changes in synaptic formation and neuronal migration, which are essential for neural adaptation. In the setting of AWS, disruptions in these synaptic processes could contribute significantly to the development and severity of withdrawal symptoms. This study aims to illuminate possible genetic interplay, examining SNPs in each gene individually as well as analyzing combinations of variants across the two loci. We hypothesize that the aggregated small effects of functional polymorphisms affecting complementary neuronal mechanisms could accumulate to exert a more pronounced genetic influence on anxiety and aggression severity of AWS. Therefore, the objective of this study was to examine the effects of *ANK3* and *ZNF804A* variants, as well as their interaction, on the anxiety and aggression severity of AWS in patients diagnosed with AUD.

## Method

### Participants

A total of 449 male patients diagnosed with AUD from six hospitals were included in the current study. The major inclusion criteria were as follows: (1) Diagnosis of AUD by at least two psychiatrists according to DSM-IV criteria; (2) Ability to read and write Chinese characters; (3) No medical history of other major neurological or psychiatric disorders. The exclusion criteria were as follows: (1) Diagnosis of severe cardiovascular, renal, hepatic diseases, or malignancy; (2) History of prior psychotic illness or having a first-degree relative with psychosis; (3) Inability to understand the essentials of the informed consent. In addition, a health control group consisting of 133 healthy volunteers was recruited at the same clinical site. The inclusion criteria for the control group were: (1) No diagnosis of AUD or any other psychiatric disorder as confirmed by clinical assessment; (2) Ability to read and write Chinese characters; (3) No significant medical history, including neurological disorders or severe physical illness. Exclusion criteria for the control group mirrored those of the AUD group, specifically: (1) Presence of severe cardiovascular, renal, hepatic diseases, or malignancy; (2) Personal or first-degree relative history of psychotic illness; (3) Inability to comprehend the informed consent process. The study protocol was approved by the Institutional Ethics Review Board of Inner Mongolia Medical University (YKD2015003). Participants completed questionnaires and provided blood samples, which were stored at -80 °C for subsequent DNA extraction. All participants gave written informed consent after being notified that their blood samples would undergo genetic analysis.

Power analysis was conducted using G*Power 3.1 to estimate required sample size. Based on findings from existing research and the basis of our previous research, the observed partial η^2^ for gene-gene interactions ranges approximately from 0.01 to 0.05 [31–33]. With input parameters of effect size f = 0.14(equivalent to a partial η^2^ of approximately 0.02), α err prob = 0.05, Power = 0.80, results indicated that 403 samples were needed to provide adequate power for detecting SNP x SNP interaction effects. Thus, our analyzed sample of 449 AUD patients exceeded the minimum requirement to obtain sufficient statistical power for robust hypothesis testing of the proposed genetic relationships.

### Measures

#### Demographic information

Demographic information, including gender, age, years of education, marital status, and living status, was collected through self-administered questionnaires. All questionnaire tests were completed within one week of admission.

#### Anxiety

Anxiety status was assessed using the Zung Self-Rating Anxiety Scale (SAS), which comprises 20 items measured on a 4-point Likert scale. The scale is designed to evaluate respondents’ subjective symptoms of anxiety. Each item is rated on a scale ranging from 1 (“little or none of the time”) to 4 (“most of the time”), with higher scores indicating greater levels of anxiety. The scale demonstrates good reliability and validity in current study, with a Cronbach’s alpha of 0.864. It has been extensively utilized in substance use disorder research [34].

#### Aggression

Aggression was measured using the Chinese version of the Buss-Perry Aggression Questionnaire (BPAQ). The BPAQ evaluates aggressive tendencies as a personality trait through questionnaire items and has demonstrated good to excellent psychometric properties, with a Cronbach’s alpha of 0.80 [35]. BPAQ is well-established in the literature for assessing aggression, including in research in the area of substance use [34, 36].

### DNA collection and extraction

Genomic DNA was extracted from 5 mL of peripheral blood using the salting-out method. Genotyping of *ANK3* rs10994336 and *ZNF804A* rs7597593 was performed using the 5’ nuclease fluorescent TaqMan™ primer (Applied Biosystems, Foster City, CA) [37]. 10% of the samples were randomly selected and tested, and no genotype errors were detected.

### Statistical analyses

Firstly, Hardy-Weinberg equilibrium (HWE) was assessed using the chi-square test. Additionally, independent samples t-tests were conducted comparing AUD patients and healthy controls on withdrawal symptom severity measured by the BPAQ and SAS scores. Chi-square tests were also performed to examine potential differences in genotype distributions between groups. Then, independent t-tests were conducted to examine the effects of single-nucleotide variants in *ANK3* rs10994336 or *ZNF804A* rs7597593 on BSAQ and SAS scores. Subsequently, a multivariate analysis of variance (MANOVA) was performed to explore allelic effects, allowing for the simultaneous detection of the main effect of a single gene and the interaction effect between genes. Next, a 2 × 2 analysis of variance (ANOVA) was conducted to determine the significance of univariate effects with two between-subjects factors: *ANK3* rs10994336 (CC homozygote vs. T allele carriers) and *ZNF804A* rs7597593 (CC homozygote vs. T allele carriers). To avoid type I errors, univariate effects within MANOVA were examined only when the overall MANOVA was significant [38]. Interactions that showed statistical significance were further analyzed using simple main effects analysis and planned comparisons to identify specific patterns of allelic effects. The analyses of simple main effects were planned, orthogonal, and therefore did not require controlling for multiple comparisons [39].

Additionally, a series of hierarchical regression analyses were conducted to examine the specific and interaction effects of *ANK3* rs10994336 and *ZNF804A* rs7597593 on aggression and anxiety. The hierarchical regression analysis consisted of two steps. In the first step, *ANK3* and *ZNF804A* were entered as predictors in the regression model to confirm the main effect of each gene. In the second step, the two-way interaction term of *ANK3* and *ZNF804A* was included in the model to test whether the effect of *ANK3* on aggression and anxiety varied depending on *ZNF804A*.

## Result

### Demographics and genotypic results

A total of 449 AUD patients and 133 healthy controls participants were included in the current study. All AUD patients were male, ranging in age from 20 to 67 years old, with a mean age of 40.29 ± 10.14 years. The average number of years of education was 11.45 ± 2.80 years, with a range of 5 to 17 years. In terms of living conditions, 76.6% of the participants lived with their families, and the proportion of married participants was 70.2%, as shown in Table 1. The basic information of the 133 healthy controls is also shown in Table 1. The results of the independent sample t test showed that anxiety, physical aggression, verbal aggression, anger and hostility in AUD patients during the withdrawal period were significantly higher than those in the control group (*p* < 0.05). In terms of emotion, the average SAS score for AUD patients was 32.84 ± 9.23. Regarding BPAQ, among AUD patients the highest scores were observed for physical aggression (*M* = 33.81, *SD* = 21.30), anger (*M* = 33.57, *SD* = 23.72), and verbal aggression (*M* = 33.21, *SD* = 20.21), while the lowest scores were observed for hostility (*M* = 26.38, *SD* = 19.08).

The genotype frequencies of both *ANK3* rs10994336 and *ZNF804A* rs7597593 were consistent with Hardy-Weinberg equilibrium (all *p* > 0.05). Table 2 presents detailed genotype frequencies and HWE *p*-values for each group. Moreover, Chi-square test showed that there was no significant difference in SNP distribution frequency between the AUD patients and healthy controls (*ANK3* rs10994336: *χ*^2^ = 1.01, *p* = 0.60; *ZNF804A* rs7597593: *χ*^2^ = 1.52, *p* = 0.47).

**Table 1:** Demographic information for full sample

	Health control (*n* = 133)	AUD patients (*n* = 449)	t	*p*	Cohen’s d
Age	37.38 ± 8.56	40.29 ± 10.14	3.01	0.003	0.30
Educational years	12.00 ± 2.62	11.45 ± 2.80	2.03	0.04	0.20
Anxiety	29.47 ± 8.54	32.84 ± 9.23	3.76	< 0.001	0.37
Physical aggression	28.46 ± 17.49	33.81 ± 21.30	2.64	< 0.001	0.26
Verbal aggression	27.74 ± 16.66	33.21 ± 20.21	2.84	< 0.001	0.28
Anger	23.40 ± 18.54	33.57 ± 23.72	4.55	< 0.001	0.45
Hostility	18.26 ± 14.03	26.38 ± 19.08	4.55	< 0.001	0.45

**Table 2:** Hardy-Weinberg equilibrium

	Major allele frequency			χ^2^	*p*
AUD patients	major allele homozygous	heterozygous	minor allele		
*ANK3* rs10994336	CC 261(58.1%)	CT 161(35.9%)	TT 27(6.0%)	0.11	0.744
*ZNF804A* rs7597593	CC 173(38.5%)	CT 202(45.0%)	TT 74(16.5%)	1.32	0.250
Health control					
*ANK3* rs10994336	CC 72(54.1%)	CT 54(40.6%)	TT 7(5.3%)	0.59	0.441
*ZNF804A* rs7597593	CC 59(44.4%)	CT 53(39.8%)	TT 21(15.8%)	2.32	0.127

### Single-gene effects

First, the correlation between withdrawal symptoms was analyzed using Pearson’s correlation analysis. Spearman correlation analysis was performed to evaluate the correlations between the genotypes of *ZNF804A* and *ANK3* and the severity of withdrawal symptoms, as shown in Table 3. The results of correlation analyses showed a strong correlation between different withdrawal symptoms. Specifically, anxiety during withdrawal was significantly associated with different forms of aggression (physical aggression: *r* = 0.30, *p* < 0.001; verbal aggression: *r* = 0.31, *p* < 0.001), particularly with anger (*r* = 0.42, *p* < 0.001) and hostility (*r* = 0.41, *p* < 0.001). Genetically, the results indicated that *ANK3* rs10994336 was significantly correlated with verbal aggression (*r* = 0.11, *p* = 0.024), anger (*r* = 0.13, *p* = 0.006), and hostility (*r* = 0.10, *p* = 0.040). However, there were no significant relationships between *ZNF804A* rs7597593 and anxiety or different forms of aggression (|*r*| < 0.03, *p* > 0.121).

Independent samples t-tests were conducted to examine the effects of single-gene mutations in *ANK3* or *ZNF804A* on anxiety and aggression, as shown in Table 4. The results revealed a significant effect of *ANK3* rs10994336 on aggression in male alcohol-dependent patients. Specifically, alcohol-dependent patients carrying the *ANK3* rs10994336 T allele exhibited higher levels of verbal aggression (*t* = -2.27, *p* = 0.024), anger (*t* = -2.76, *p* = 0.006), and hostility (*t* = -2.06, *p* = 0.040). *ZNF804A* rs7597593 had no significant effect on anxiety (*t* = 1.36, *p* = 0.175) or different forms of aggression (*ts* < 1.55, *ps* > 0.121).

**Table 3:** Correlations of *ANK3* and ZNF804A genotypes with anxiety and aggression

	1.	2.	3.	4.	5.	6.	7.
1.*ANK3*	1						
2.*ZNF804A*	0.04	1					
3.Anxiety	0.07	-0.05	1				
4.Physical aggression	0.05	-0.06	0.30^***^	1			
5.Verbal aggression	0.11^*^	-0.03	0.31^***^	0.68^***^	1		
6.Anger	0.13^*^	-0.05	0.42^***^	0.67^***^	0.76^***^	1	
7.Hostility	0.10^*^	-0.07	0.41^***^	0.60^***^	0.71^***^	0.67^***^	1

Note. *p < 0.05, **p < 0.01 ***p < 0.001

**Table 4:** The single effects of *ANK3* and *ZNF804A* polymorphisms on anxiety and aggression

	ANK3 rs10994336					ZNF804A rs7597593			
	*ANK3*: CC homozygote (*n* = 261)	*ANK3*: T allele (*n* = 188)	*t*	*p*		*ZNF804A*: CC homozygote (*n* = 173)	*ZNF804A*: T allele (*n* = 276)	*t*	*p*
Anxiety	32.30 ± 8.98	33.61 ± 9.54	-1.49	0.14		33.36 ± 9.95	32.52 ± 8.75	-0.95	0.35
Physical aggression	32.81 ± 21.30	35.14 ± 21.29	-1.13	0.26		35.53 ± 22.16	32.73 ± 20.72	-1.36	0.18
Verbal aggression	31.38 ± 19.89	35.74 ± 20.44	-2.27	0.02		33.99 ± 22.13	32.72 ± 18.94	-0.65	0.52
Anger	30.97 ± 22.63	37.19 ± 24.77	-2.76	0.01		35.02 ± 24.10	32.67 ± 23.48	-1.02	0.31
Hostility	24.81 ± 18.65	28.55 ± 19.49	-2.06	0.04		28.14 ± 20.29	25.27 ± 18.22	-1.55	0.12

### Allelic group

**Fig. 1:**
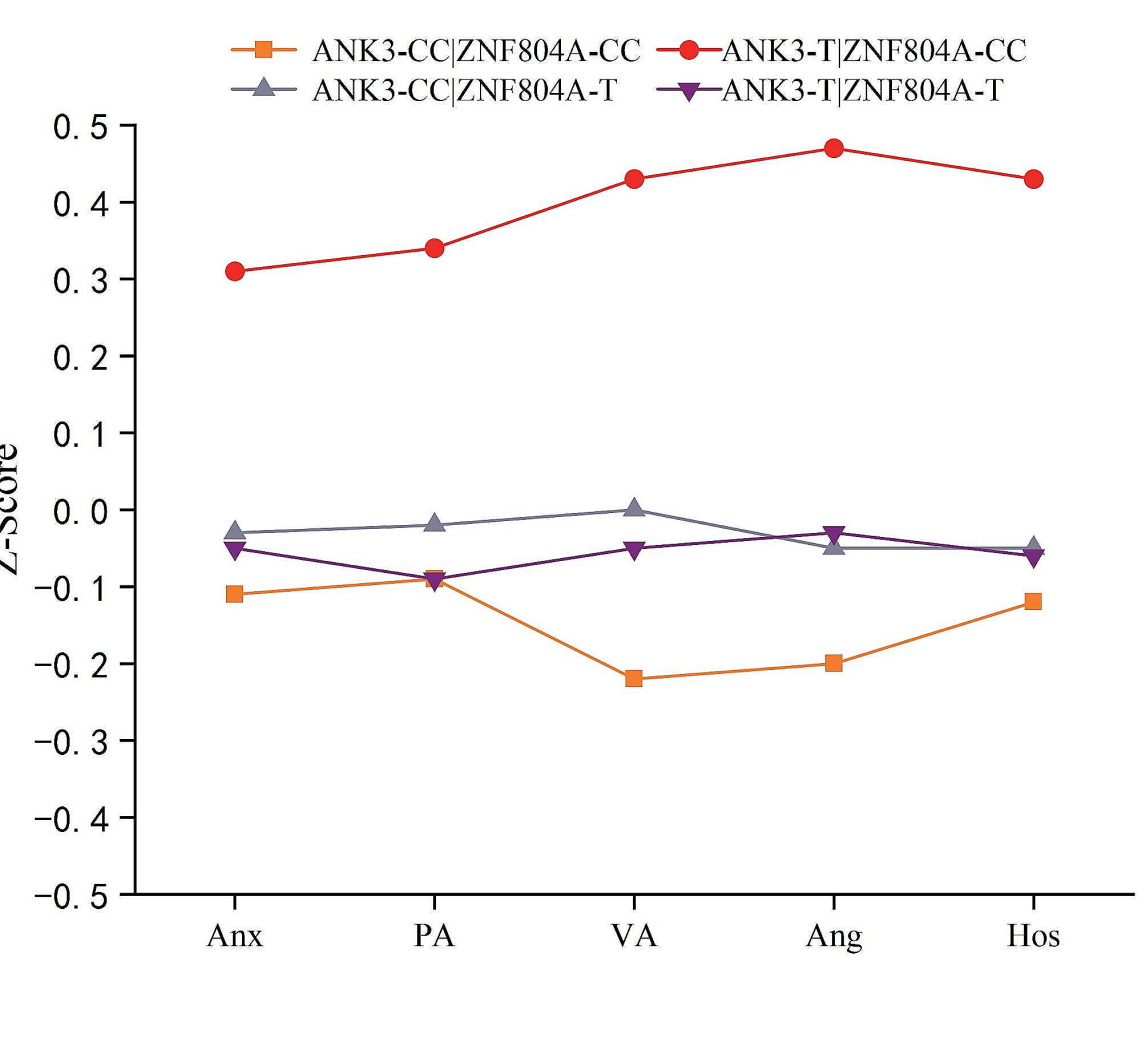
Standard normalized score of Anxiety (Anx), Physical Aggression (PA), Verbal Aggression (VA), Anger (Ang) and Hostility (Hos) for the four allelic groups

Figure 1 depicts the scores for anxiety and different forms of aggression among the four allelic subgroups. As shown in Fig. 1, individuals carrying the *ANK3* rs10994336 T allele and *ZNF804A* rs7525957 CC homozygote genotype had the highest anxiety and aggression scores. They were followed by individuals with the *ANK3* rs10994336 CC homozygote and *ZNF804A* rs7525957 T allele genotype, and then by individuals with the *ANK3* rs10994336 T allele and *ZNF804A* rs7525957 T allele genotype. The lowest anxiety and aggression scores were observed in individuals with the *ANK3* rs10994336 CC homozygote and *ZNF804A* rs7525957 CC homozygote genotype.

To examine the allelic group effect, a multivariate analysis of variance (MANOVA) was performed, considering anxiety, physical aggression, verbal aggression, anger, and hostility. The MANOVA with two between-subjects factors, *ANK3* rs10994336 and *ZNF804A* rs7597593, revealed a significant interaction (*F*_(5, 441)_ = 2.99, *p* = 0.012, Partial Eta Squared = 0.033). Subsequently, univariate analysis of variance (ANOVA) with *ANK3* rs10994336 (2 levels) × *ZNF804A* rs7597593 (2 levels) separately revealed significant interaction effects on anxiety (*F*_(1, 445)_ = 5.06, *p* = 0.025, Partial Eta Squared = 0.011) and the four different forms of aggression (*Fs*_(1, 445)_ > 6.69, *ps* < 0.010, Partial Eta Squared > 0.015) more details are listed in Table 5.

**Table 5:** The interaction effects of *ANK3* and *ZNF804A* polymorphisms on anxiety and aggression

Parameter	Factor	SS	Df	MS	F	*P*	η^2^*p*
Anxiety	*ANK3* rs10994336	349.04	1	349.04	4.15	0.042	0.009
	*ZNF804*A rs7597593	166.56	1	166.56	1.98	0.160	0.004
	Int	426.44	1	426.44	5.06	0.025	0.011
	Residual	37470.29	445	84.202			
Physical Aggression	*ANK3* rs10994336	1431.99	1	1431.99	3.21	0.074	0.007
	*ZNF804A* rs7597593	1551.75	1	1551.75	3.47	0.063	0.008
	Int	2989.91	1	2989.91	6.69	0.010	0.015
	Residual	198821.81	445	446.79			
Verbal Aggression	*ANK3* rs10994336	3866.21	1	3866.21	9.80	0.002	0.022
	*ZNF804A* rs7597593	769.84	1	769.84	1.95	0.163	0.004
	Int	5084.13	1	5084.13	12.88	< 0.001	0.028
	Residual	175640.12	445	394.70			
Anger	*ANK3* rs10994336	6933.38	1	6933.38	12.80	< 0.001	0.028
	*ZNF804A* rs7597593	1669.05	1	1669.05	3.08	0.080	0.007
	Int	6214.05	1	6214.05	11.47	0.001	0.025
	Residual	240995.12	445	541.56			
Hostility	*ANK3* rs10994336	2736.87	1	2736.87	7.73	0.006	0.017
	*ZNF804A* rs7597593	1641.30	1	1641.30	4.64	0.032	0.010
	Int	2914.38	1	2914.38	8.23	0.004	0.018
	Residual	157595.27	445	354.15			

Note: Int = *ANK3* rs10994336 × *ZNF804A* rs7597593

Simple main effects analysis and multiple comparisons further explore the specifics of the interaction. The analysis indicates that the simple main effect of *ANK3* rs10994336 genetic variation on anxiety only exists in *ZNF804A* rs7525957 CC homozygote carriers (*F*_(1, 445)_ = 7.39, *p* = 0.007, Partial Eta Squared = 0.016). Specifically, in *ZNF804A* rs7525957 CC homozygote carriers, *ANK3* rs10994336 T allele carriers (*M* = 31.84, *SD* = 9.57) exhibit higher anxiety than CC homozygote carriers (*M* = 35.72, SD = 10.14) (*t* = 2.72, *p* = 0.007). However, in *ZNF804A* rs7525957 T allele carriers, there is no significant simple main effect of *ANK3* rs10994336 genetic variation on anxiety (*F*_(1, 445)_ = 0.03, *p* = 0.862, Partial Eta Squared < 0.001). *ANK3* rs10994336 T allele carriers show no significant difference in anxiety compared with CC homozygote carriers (*t* = -0.17, *p* = 0.862). The same effect is observed for aggression, where the simple main effect of *ANK3* rs10994336 genetic variation on aggression only exists in *ZNF804A* rs7525957 CC homozygote carriers (*Fs*_(1, 445)_ > 7.70, *ps* < 0.006, Partial Eta Squared > 0.017). In *ZNF804A* rs7525957 CC homozygote carriers, *ANK3* rs10994336 T allele carriers exhibit higher anxiety than CC homozygote carriers (*t* = 2.78, *p* = 0.006). These results suggest that the *ANK3* rs10994336 T allele acts as a variant (risk) allele whose effect is regulated by another gene, *ZNF804A* rs7525957. Specifically, the simple main effect of *ANK3* rs10994336 variation only exists in *ZNF804A* rs7525957 CC homozygote carriers, and *ZNF804A* rs7525957 antagonizes the effect of the *ANK3* rs10994336 T allele variation. Regarding ZNF804A rs7525957, the antagonistic effects are only present in *ANK3* rs10994336 T allele (risk allele) carriers (*Fs*_(1, 445)_ > 5.66, *ps* < 0.018, Partial Eta Squared > 0.013). For *ANK3* rs10994336 CC homozygote carriers, *ZNF804A* rs7525957 T allele carriers do not show lower anxiety and aggression compared to CC homozygote carriers. Therefore, the moderating effect of *ZNF804A* rs7525957 on anxiety and aggression appears only in *ANK3* rs10994336 T allele variation.

**Fig. 2:**
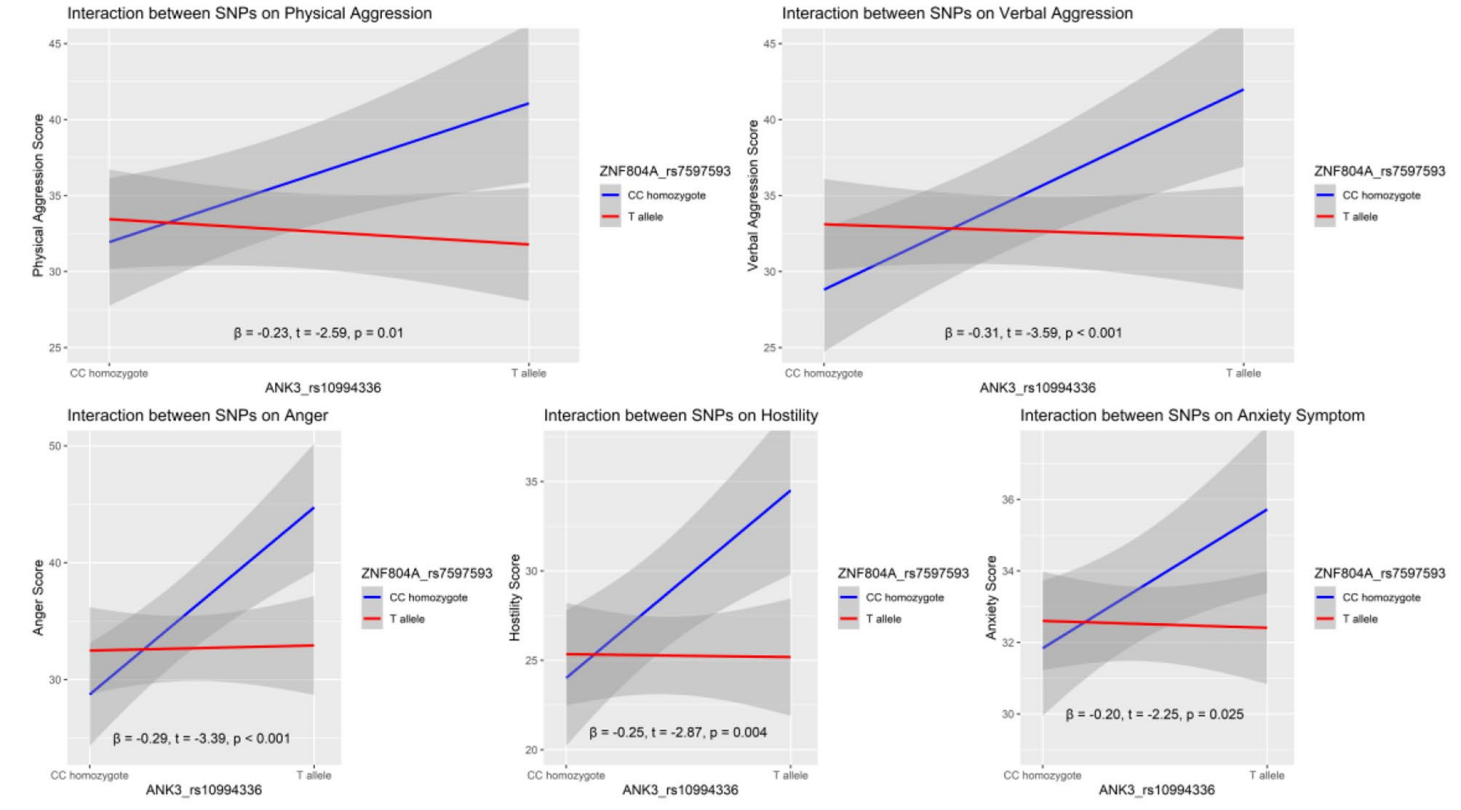
The interaction effects of *ANK3* and *ZNF804A* polymorphisms on anxiety and aggression

Lastly, a series of hierarchical regression analyses was conducted to confirm the interaction effects of *ANK3* and *ZNF804A* on anxiety and aggression. *ANK3*, *ZNF804A*, and the interaction term *ANK3* x *ZNF804A* were entered as predictors of anxiety and aggression. The interaction of *ANK3* and *ZNF804A* accounted for a significant portion of the variance in anxiety (*β* = -0.20, *t* = -2.25, *p* = 0.025) and aggression (|*β*|s > 0.23, |*t*|s > 2.59, *p* < 0.010). The partial correlation for this interaction in anxiety was − 0.11, indicating that approximately 1.2% of the variance in anxiety could be explained by the *ANK3*-*ZNF804A* interaction. The partial correlation for this interaction in aggression ranged from − 0.12 to -0.17, suggesting that approximately 1.4–2.8% of the variance in aggression could be explained by the *ANK3*-*ZNF804A* interaction (More details of different forms of aggression are shown in Fig. 2).

### Specific analyses in healthy controls

The present study utilized interaction analysis to examine the effect of *ANK3* and *ZNF804A* on AWS-related symptoms. To determine the specificity of this gene × gene effect, identical analyses were performed on a group of healthy controls, but no significant results were found.

Table 2 displays the genotype frequencies in the healthy control group, where the genotype frequencies of *ANK3* rs10994336 and *ZNF804A* rs7597593 were in Hardy-Weinberg equilibrium (*ANK3*: *χ2* = 0.59, *p* = 0.441; ZNF804A: *χ2* = 2.32, *p* = 0.127). The results of MANOVA, which examined the interaction gene effect, demonstrated that none of the genetic interactions mentioned above had a significant impact on the psychiatric symptoms in healthy controls (*F*_(5, 125)_ = 0.572, *p* = 0.721, Partial Eta Squared = 0.022. The ANOVA results further indicated that in healthy controls, *ANK3* rs10994336 and *ZNF804A* rs7597593 had no significant interaction effect on multiple psychiatric symptoms (*Fs*_(1, 125)_ < 0.90, *ps* > 0.346, Partial Eta Squared < 0.007). The complete ANOVA results, including interactions, are presented in supplementary Table S1.

## Discussion

In this study, we have demonstrated that the severity of withdrawal symptoms in AUD patients is influenced by the interaction effects of genetic polymorphisms from two distinct genes, *ANK3* rs10994336 and *ZNF804A* rs7597593. This interaction reveals a novel function of *ANK3* and *ZNF804A* in jointly modulating neuronal activity during the acute withdrawal phase. Specifically, our findings indicate that the presence of the *ANK3* rs10994336 T variant allele is associated with increased severity of AWS symptoms, including verbal aggression, anger, and hostility during the withdrawal phase. Importantly, this risk factor is regulated by the interaction with regulatory genes, particularly the *ZNF804A* rs7597593 T variant allele, which appears to mitigate the exacerbation of psychiatric symptoms associated with the *ANK3* rs10994336 T allele variant. It is worth noting that *ZNF804A* rs7597593 CC homozygotes did not exhibit the same effect.

First, regarding the *ANK3* gene, the single-SNP analysis demonstrated that the *ANK3* rs10994336 T allele, as a risk variant, is associated with the severity of AWS. This result is consistent with studies on schizophrenia and bipolar disorder, which have linked the T allele variant to psychotic symptoms [40–42]. Neuroimaging studies have reported that carriers of the T allele of *ANK3* rs10994336 exhibit reduced fractional anisotropy, indicating less strict alignment of axonal fibers and impaired connections between subcortical and frontal regions. This suggests that the risk variant T allele of rs10994336 could influence white matter integrity and alter neural circuits [21, 43]. Several neuropsychological findings also support this notion, indicating that the T allele impacts cognition by impairing sustained attention [44], altering decision-making [45], and increasing risk-taking behavior [21]. The evidence aligns with the outcomes of this study, where AUD patients with the CT/TT genotype of rs10994336 scored higher on measures of anger, hostility, and verbal aggression compared to those with the CC genotype during alcohol withdrawal. This suggests that the T allele may exacerbate alcohol withdrawal syndrome in patients with AUD. Overall, the evidence presented indicates a potential functional effect of rs10994336 on AWS-related mood and behavior. Recently, Roussos et al. reported that the molecular mechanism of *ANK3* genetic susceptibility to mental disorders may involve reduced *ANK3* gene and protein expression [46]. However, the polymorphism rs10994336, located in the intron of the *ANK3* gene, has not yet revealed its exact effect on *ANK3* mRNA expression in the brain. Therefore, further investigation is needed to understand the molecular mechanism through which rs10994336 regulates the severity of AWS.

Second, in the current study, the results reveal a statistically significant gene-gene interaction between *ANK3* and *ZNF804A* on AWS. This interaction indicates that the severity of AWS, as measured by psychiatric symptoms including mood and behavior, systematically varies depending on the alleles of both *ANK3* and *ZNF804A*. The single-SNP analysis showed that *ZNF804A* rs7597593 has no significant association with the severity of AWS. However, MANOVA and a series of interaction analyses revealed that *ZNF804A* rs7597593 could regulate the effect of *ANK3* rs10994336 on AWS-related mood and behavior, including anxiety, physical and verbal aggression, anger, and hostility. In other words, these results suggest that the *ANK3* rs10994336 T allele may have a risk-enhancing effect when paired with the *ZNF804A* rs7597593 CC homozygote, but this risk-enhancing effect disappears when coupled with the *ZNF804A* rs7597593 T allele. However, the *ANK3* rs10994336 CC homozygote, regardless of whether paired with the *ZNF804A* rs7597593 CC homozygote or *ZNF804A* rs7597593 T allele, does not show a risk-enhancing effect.

The interaction between ANK3 rs10994336 and ZNF804A rs7597593 polymorphisms may have significant implications for the neurobiological mechanisms underlying AWS. ANK3 plays a pivotal role in regulating voltage-gated sodium channels, which are essential for action potential generation and propagation in neurons [16]. ZNF804A, on the other hand, is implicated in synaptic plasticity and neural connectivity, influencing gene expression patterns related to neuronal signaling [14]. The interaction between ANK3 and ZNF804A genes can modulated the crosstalk between neuronal signaling pathways, which may result in changes in neuronal excitability and synaptic transmission. These alterations could contribute to the pathophysiology of AWS. Additionally, this genetic interaction may cause structural changes in synaptic connections, which could impact the effectiveness of neural circuits involved in stress response and emotional regulation [47]. Moreover, the complex genetic interaction between ANK3 and ZNF804A polymorphisms could have significant effects on neurotransmitter systems, particularly those that involve gamma-aminobutyric acid (GABA) and glutamate. These neurotransmitters are crucial in modulating neuronal excitability and the pathophysiology of AWS. ANK3 has a regulatory role on ion channels and membrane dynamics, which can significantly impact the GABAergic system. This has the potential to alter inhibitory neurotransmission, thereby modulating neuronal stability and excitability [48, 49]. Concurrently, ZNF804A, known for its involvement in synaptic plasticity and neural connectivity, which may influence glutamatergic pathways. This can affect excitatory neurotransmission and neural circuit functionality [47, 50]. The genetic variations in ANK3 and ZNF804A may work together to disrupt the balance between excitatory and inhibitory neurotransmission, leading to exacerbated AWS symptoms through enhanced neural excitability and dysregulated stress and emotion regulation pathways. This speculation is supported by emerging evidence that alterations in GABAergic and glutamatergic systems are central to the neurobiological mechanisms of AWS [51].

The potential role of ANK3 and ZNF804A variants in modulating mood and behavior in the context of AWS is underscored by their synergistic effects on neural circuits, particularly in the amygdala and prefrontal cortex in the context of AWS [52]. The amygdala, a key region implicated in anxiety and fear processing, and the prefrontal cortex, central to executive functions and emotional regulation, are both crucial in the neurobiological underpinnings of AWS. Variants in the ANK3 gene may influence the structural and functional integrity of neural circuits by affecting axonal stability and synaptic organization, which can influence the transmission of emotional signals [48]. Concurrently, ZNF804A variants have been linked to changes in gene expression patterns that regulate synaptic plasticity and neural connectivity, resulting in alterations in the functional dynamics between the amygdala and prefrontal cortex. The interaction between these genetic variations have a compounded effect on neural circuits [53, 54], potentially leading to dysregulated emotional responses and heightened susceptibility to anxiety and aggression, hallmark symptoms of AWS. Neuroimaging studies further support this notion, revealing that individuals carrying these genetic variants exhibit altered connectivity patterns between the amygdala and prefrontal cortex. These patterns are associated with increased susceptibility to mood disorders [55]. Understanding the interaction by which ANK3 and ZNF804A variants influence neural circuits provides valuable insights into the genetic architecture of AWS and highlights the importance of exploring these genetic interactions in the development of targeted therapeutic interventions. Recent genome-wide association studies have revealed the effect of mutations in *ANK3* and *ZNF804A* on brain white matter integrity [56–58]. In those studies, both diffusion tensor imaging (DTI) and Tract-Based Spatial Statistics (TBSS) results show that *ANK3* and *ZNF804A* risk alleles are associated with lower fractional anisotropy (FA) of white matter in multiple brain regions. Although the neural mechanism of the interaction between these two genes is still unclear, both genes modulate brain white matter integrity and nerve conduction, and both are closely related to the dopamine system, which might be the physiological basis of their interaction on psychiatric symptoms.

Taken together, the most striking and novel findings from this study point to the strong association of naturally occurring allelic variants in *ANK3* rs10994336 (CC homozygous vs. T allele carriers) with AWS-related mood and behavior, where the T allele is the risk allele. Additionally, these findings suggest a protective role of the *ZNF804A* rs7597593 T allele against the *ANK3* rs10994336 mutation, with this allele mitigating, if not reversing, the risk effects of the *ANK3* rs10994336 T allele. The current results provide tentative support for gene-gene interaction in explaining psychiatric risk, which may enhance our understanding of the pathophysiological mechanisms underlying AWS. In the future, *ANK3*-*ZNF804A* interaction analysis should be investigated in other psychiatric disorders, such as bipolar disorder and schizophrenia, which have been found to be highly correlated with the *ANK3* or *ZNF804A* gene [53, 59, 60].

### Limitation

Finally, the current study has several limitations that should be addressed in future research. Firstly, the underlying mechanism to explain these results was not identified in the present study. Therefore, future studies should investigate the expression changes of these two genes in carriers of different alleles and analyze protein interaction networks or shared signaling pathways to uncover the mechanisms underlying the regulation of psychiatric symptoms by these two genes.

Additionally, the sample size of the present study, while reasonable, consisted only of male alcohol use patients and male healthy controls. On one hand, this resulted in a high degree of homogeneity in the study population, but on the other hand, it complicates the generalizability of the findings to the broader population. Therefore, future studies should include a larger sample size and consider participants of different genders to replicate the present findings.

Furthermore, this study only examined AWS-related aggression and anxiety, lacking relevant physiological data. Although recent studies provide evidence of physiological changes associated with the rs10994336 T risk gene [15, 61], future studies should build upon the present findings by further exploring the physiological changes that occur under the interaction of *ANK3* and *ZNF804A*.

### Electronic supplementary material

Below is the link to the electronic supplementary material.

Supplementary Material 1

## Data Availability

The datasets presented in this article are not readily available due to the inclusion of student-specific information. Requests to access the datasets should be directed to *shenguanghuipsy@163.com* or wangwei@wmu.edu.cn.

## Declarations

All procedures were performed in accordance with relevant guidelines from declaration of Helsinki statement.
