# Supplementary material for: ANK3 rs10994336 and ZNF804A rs7597593 polymorphisms: genetic interaction for emotional and behavioral symptoms of alcohol withdrawal syndrome
Source: BMC Psychiatry. 2024 May 3;24:335. doi: 10.1186/s12888-024-05787-z (PMC11067186; doi:10.1186/s12888-024-05787-z)
Supplement: Supplementary file 1 — Supplementary Material 1 [file 12888_2024_5787_MOESM1_ESM.docx]

**Supplementary Material**

Table S1 The interaction effects of *ANK3* and *ZNF804A* polymorphisms in healthy controls

| Parameter | Factor | *SS* | *Df* | *MS* | *F* | *P* | *η*^2^*p* |
| --- | --- | --- | --- | --- | --- | --- | --- |
| Anxiety | *ANK3* rs10994336 | 76.25 | 1 | 76.25 | 1.05 | 0.307 | 0.008 |
|  | *ZNF804A* rs7597593 | 156.78 | 1 | 156.78 | 2.16 | 0.144 | 0.016 |
|  | Int | 64.96 | 1 | 64.96 | 0.90 | 0.346 | 0.007 |
|  | Residual | 9394.16 | 129 | 72.82 |  |  |  |
| Physical Aggression | *ANK3* rs10994336 | 398.47 | 1 | 398.47 | 1.30 | 0.256 | 0.010 |
|  | *ZNF804A* rs7597593 | 345.02 | 1 | 345.02 | 1.13 | 0.290 | 0.009 |
|  | Int | 107.24 | 1 | 107.24 | 0.35 | 0.555 | 0.003 |
|  | Residual | 39442.64 | 129 | 305.76 |  |  |  |
| Verbal Aggression | *ANK3* rs10994336 | 203.35 | 1 | 203.352 | 0.74 | 0.393 | 0.06 |
|  | *ZNF804A* rs7597593 | 468.08 | 1 | 468.08 | 1.69 | 0.196 | 0.013 |
|  | Int | 239.56 | 1 | 239.56 | 0.87 | 0.354 | 0.007 |
|  | Residual | 35695.11 | 129 | 276.71 |  |  |  |
| Anger | *ANK3* rs10994336 | 18.70 | 1 | 18.70 | 0.06 | 0.816 | < 0.001 |
|  | *ZNF804A* rs7597593 | 1092.75 | 1 | 1092.75 | 3.19 | 0.077 | 0.024 |
|  | Int | 3.92 | 1 | 3.92 | 0.01 | 0.915 | < 0.001 |
|  | Residual | 44262.41 | 129 | 343.12 |  |  |  |
| Hostility | *ANK3* rs10994336 | 562.99 | 1 | 562.99 | 2.86 | 0.093 | 0.022 |
|  | *ZNF804A* rs7597593 | 35.53 | 1 | 35.53 | 1.81 | 0.671 | 0.001 |
|  | Int | 5.72 | 1 | 5.72 | 0.29 | 0.865 | < 0.001 |
|  | Residual | 25363.05 | 129 | 196.61 |  |  |  |

Note：Int = *ANK3* rs10994336 × *ZNF804A* rs7597593
